# Participants who were difficult to recruit at baseline are less likely to complete a follow-up questionnaire – results from the German National Cohort

**DOI:** 10.1186/s12874-020-01073-0

**Published:** 2020-07-09

**Authors:** Stefan Rach, Kathrin Günther, Birte Hadeler

**Affiliations:** Leibniz Institute for Prevention Research and Epidemiology – BIPS, Achterstrasse 30, 28359 Bremen, Germany

**Keywords:** Epidemiologic methods, Surveys and questionnaires, Lost to follow-up, Follow-up studies, German National Cohort

## Abstract

**Background:**

With declining response proportions in population-based research the importance of evaluating the effectiveness of measures aimed at improving response increases. We investigated whether an additional flyer with information about the study influences participation in a follow-up questionnaire and the time participants take to send back filled questionnaire.

**Methods:**

In a trial embedded within the German National Cohort we compared responses to invitations for a follow-up questionnaire either including a flyer with information about the cohort study or not including it. Outcomes of interest were participation in the follow-up (yes vs. no) and time to response (in days). We analyzed paradata from baseline recruitment to account for differences in recruitment history between participants.

**Results:**

Adding a flyer to invitations did neither influence the likelihood of participation in the follow-up (OR 0.94, 95% CI: 0.80, 1.11), nor the time it took participants to return completed questionnaires (β̂ = 1.71, 95% CI: − 1.01, 4.44). Subjects who, at baseline, needed to be reminded before eventually participating in examinations and subjects who scheduled three or more appointments until eventually completing baseline examinations were less likely to complete the follow-up questionnaire and, if they did, took more time to complete questionnaires.

**Conclusions:**

Evaluating the effectiveness of measures aimed at increasing response can help to improve the allocation of usually limited resources. Characteristics of baseline recruitment can influence response to follow-up studies and therefore information about recruitment history (i.e., paradata) might prove useful to tailor follow-up recruitments to those who were difficult to recruit during baseline. To this end, however, it is necessary to routinely and meticulously collect paradata during recruitment.

## Background

Population-based research is increasingly challenged by decreasing response proportions [1–4], threatening the generalizability of studies due to possibly biased estimates [5, 6]. It has been shown that already technical details of the delivery can influence participation [6] and, consequently, it is important to evaluate whether measures taken to increase response indeed accomplish the desired effect and/or whether previous results transfer to other cultural contexts or across social changes over time. In this trial conducted within the German National Cohort (GNC, German: NAKO Gesundheitsstudie [7]), we investigated whether an additional flyer with information about the study influences participation in a follow-up questionnaire and the time participants take to send back the filled questionnaire.

An additional challenge for longitudinal studies is that participants who were more difficult to recruit initially (so called late respondents) are more likely to drop out in later stages of the study [8–11], that is, additional efforts spent at baseline to increase response and representativeness are possibly not rewarded at follow-ups. To investigate whether characteristics of recruitment during baseline influenced participation in the follow-up questionnaire, our analyses also included explanatory variables derived from paradata, i.e., detailed information about the recruitment process [12] (see methods for details).

## Methods

The GNC is a cohort study investigating causes of major chronic diseases that is conducted in 18 regional study centers across Germany [7]. Baseline examinations conducted from 2014 to 2019 included a total of 205,217 participants aged 20–69 randomly drawn from regional registries of residents. In the study center of Bremen, where this trial was conducted, 10,486 participants were examined. Examinations included computer-assisted personal face-to-face interviews, a number of standardized physical and medical examinations, the collection of various biomaterials, and self-completion questionnaires (“Level 1” protocol). A random sub-sample of 20% completed an extended protocol including more in-depth physical and medical examinations (“Level 2” protocol). All participants will be re-invited to a re-assessment after 4–5 years. In addition, all participants will be re-contacted every 2–3 years and asked to fill in questionnaires about changes in lifestyle, the occurrence of diseases, and other characteristics. A detailed study protocol can be found elsewhere [7, 13], as well as a detailed description of the recruitment protocol during baseline [14].

All procedures performed in the GNC were in accordance with the ethical standards of the institutional and/or national research committee and with the 1964 Helsinki declaration and its later amendments or comparable ethical standards. The study was approved by the Ethics committee of the local chamber of physicians in Bremen (Bremen Medical Association, reference number RE/HR-388). Written informed consent was obtained from all participants included in the GNC.

The current trial ran from April 2018 to February 2019 within the first round of follow-up questionnaires in Bremen’s study center. Participants were invited to fill a 16-pages paper & pencil questionnaire inquiring about, for instance, their general health status, height and weight, selected disease symptoms, use of medication, smoking, menopausal status, and the occurrence of diseases (diagnosed by a physician). Invitations were sent by traditional mail and included a pre-stamped return envelope, which participants could use to return the questionnaire. If a person did not respond within three weeks, a reminder letter was sent, after another two weeks followed by up to five phone attempts over a span of four weeks, and, finally, a second reminder letter was sent. The recruitment was controlled and documented with MODYS, a dedicated software for epidemiological field studies and paradata collection (for a detailed description see [15]). MODYS schedules recruitment tasks according to a predefined recruitment protocol, provides a mail merge system to generate and print study documents (e.g., letters, invitations), and logs and time-stamps all completed actions (e.g., outbound letters and emails, passed waiting periods). In addition, the field staff uses MODYS to log and time-stamp all attempted and successful interactions with potential participants (inbound and outbound phone calls, inbound letters and emails), as well as other recruitment events (e.g., issuing of dropout codes, corrections of contact data, completion of examinations).

In addition to the questionnaire, invitations could include a leaflet (flyer) informing about the questionnaire, reporting first empirical results from baseline examinations (hand grip strength stratified by sex), informing about the olfactory function test conducted at baseline, and about reasons for the recent change of the cohort’s German name from GNC to NAKO (see additional file 1 for the German flyer and an English translations of its contents). The flyer “NAKO update” is regularly published by the public relations office of the GNC study and provided to all study centers with the encouragement to include it in any written communications with participants. The current trial investigated whether this flyer influenced response to the postal questionnaire.

A total of 3275 participants was randomized to receive an invitation either including the flyer (group *flyer*, N = 1648) or not including it (group *no-flyer*, N = 1627). To be able to detect a deviation of ±5 percentage points from the assumed base response of 75% with a power of 0.85 the sample size was set to at least N = 2938 based on a-priori power analyses (two-tailed). Participants were added to the trial in order of their invitation until the predefined sample size was reached.

Participants were eligible for this trial if they took part in the baseline examination and the examination dated back at least two years at the time of invitation (examinations between April 2014 and February 2017). Invitations were sent out according to the normal mailing schedule of the study center (usually on Tuesdays, Wednesdays, and Thursdays) and the number of invitations sent out per week was pre-determined by the number of examinations completed per week two years earlier (about 50–60 per week). Due to a backlog at the beginning of the trial, the number of invitations per week could be as high as 500 during the first weeks of the trial. Invitation letters were prepackaged by persons not involved in the day-to-day recruitment to keep regular field staff responsible for contact with participants blinded to group assignments.

**Fig. 1:**
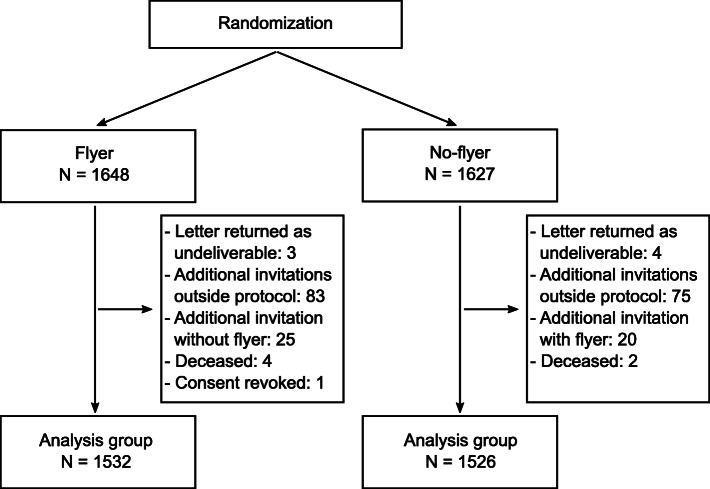
Allocation of participants

Participants were excluded from the trial if they had deceased since participating at baseline (N = 6), revoked their consent after receiving the invitation (N = 1), if letters were returned as undeliverable (N = 7), if paradata included follow-up recruitment events before the trial started (e.g., previous follow-up invitations sent to invalid addresses; N = 158), or because additional invitations were sent out mistakenly not matching their initial group assignment (N = 45). The final analysis group totaled 3058 participants (*flyer*: N = 1532; *no-flyer*: N = 1526; see Fig. 1 for a flow chart).

Outcome of interest was participation in the follow-up questionnaire (yes vs. no) for the main analysis and time to response in days for the second analysis (i.e., time between mailing of the invitation letter and return of the filled questionnaire). The outcome was assessed by the field staff involved in the day-to-day recruitment by scanning barcodes on returned questionnaires using the MODYS software, resulting in time stamped database entries. Exposure variables were invitation group (flyer vs. no-flyer), sex (female vs. male), age (categories: 20–29, 30–39, 40–49, 50–59, and ≥ 60 years), and nationality (German vs. non-German, as provided by the registry of residents). Since recruitment intensity during baseline may influence participation in follow-ups [8–11, 16] additional variables characterizing baseline recruitment were also included: number of reminder letters (0, 1, 2, ≥3), number of appointments made until eventually participating (1, 2, ≥3), availability of phone number in public phone records before baseline recruitment (yes vs. no), and study protocol completed at baseline (Level 1 vs. Level 2).

Associations were quantified by odds ratios (ORs) and 95% confidence intervals (CIs) estimated with logistic regression models for the main analysis, and by beta coefficients and 95% CIs estimated with linear regression models for the secondary analysis. To control for possible differences during the invitation process, all models were adjusted for duration between baseline examination and follow-up invitation in years and day of the week the invitation were sent. All analyses were performed using R version 3.4.3 (http://www.r-project.org/).

## Results

Out of 3058 participants included in this analysis 2226 completed the follow-up questionnaire, equaling a response proportion of 72.8% (see Table 1 for a descriptive analysis of main variables used in the study). Female persons were more frequently included in the study sample, as were persons with German nationality, reflecting that these groups are more likely to participate and therefore completed baseline recruitment earlier [17]. Elder persons were also more frequently included because the upper two age strata (50–59 and ≥ 60) were oversampled according to study design [13].

**Table 1:** Descriptive analysis of the main variables used in the study

	Group			
	No-flyer		Flyer	
	n	%	n	%
Sex				
Male	620	40.6	671	43.8
Female	906	59.4	861	56.2
Age				
20–29	153	10.0	152	9.9
30–39	280	18.3	258	16.8
40–49	268	17.6	286	18.7
50–59	376	24.6	415	27.1
≥ 60	449	29.4	421	27.5
Nationality				
German	1402	91.9	1417	92.5
Other	124	8.1	115	7.5
Examination program baseline				
Level 1	1204	78.9	1211	79.0
Level 2	322	21.1	321	21.0
Reminder baseline				
0	782	51.2	767	50.1
1	444	29.1	455	29.7
2	191	12.5	189	12.3
≥ 3	109	7.1	121	7.9
Appointments baseline				
1	1134	74.3	1094	71.4
2	287	18.8	324	21.1
≥ 3	105	6.9	114	7.4
Phone status baseline				
No phone	1225	80.3	1238	80.8
Phone	301	19.7	294	19.2
Delay (years)				
Mean (SD)	2.1	(0.3)	2.1	0.2
Weekday				
Tuesday	547	35.8	563	36.7
Wednesday	490	32.1	479	31.3
Thursday	489	32.0	490	32.0
N	1526	50.0	1532	50.0

Our analysis (Table 2) did not reveal evidence that adding a flyer to the invitation influenced the likelihood of participation in the follow-up questionnaire (OR 0.94, 95% CI: 0.80, 1.11). Female subjects were more likely to participate (OR 1.48, 95% CI: 1.26, 1.75), as were subjects in the highest age group (≥ 60) compared to subject aged 40–49 (OR 1.95, 95% CI: 1.50, 2.53). Persons with non-German nationality were less likely to take part (OR 0.33, 95% CI: 0.25, 0.44). Subjects who needed to be reminded before eventually participating in the baseline examination were less likely to complete the follow-up questionnaire (1 reminder: OR 0.68, 95% CI: 0.55, 0.84; 2 reminders: OR 0.59, 95% CI: 0.45, 0.77; ≥3 reminders: OR 0.39, 95% CI: 0.29, 0.53). Subjects who scheduled three or more appointments during baseline were less likely to participate compared to subjects completing examinations at the first scheduled appointment (OR 0.67, 95% CI: 0.49, 0.91). If the phone number of a person could be retrieved in public phone records before baseline recruitment, they were less likely to participate in the follow-up (OR 0.73, 95% CI: 0.57, 0.93).

**Table 2:** Odds ratios (95% CIs) for participating in the follow-up and β–coefficients (95% CIs) for time to response (in days) as estimated from regression models adjusted for duration between baseline examination and follow-up invitation in years and day of the week the invitation were sent

	Participation						Time to response (days)			
	No		Yes		OR^a^	(95% CI)				
	n	%	n	%			Mean	sd	β̂^a^	(95% CI)
Group										
No-flyer	404	48.6	1122	50.4	–		29.2	31.8	–	
Flyer	428	51.4	1104	49.6	0.94	(0.80, 1.11)	30.7	34.5	1.71	(−1.01, 4.44)
Sex										
Male	410	49.3	881	39.6	–		30.7	35.4	–	
Female	422	50.7	1345	60.4	1.49	(1.26, 1.75)	29.4	31.7	−0.80	(−3.59, 1.99)
Age										
20–29	100	12.0	205	9.2	1.05	(0.76, 1.41)	35.4	36.8	3.85	(−1.77, 9.47)
30–39	173	20.8	365	16.4	1.08	(0.79, 1.34)	33.0	35.6	1.62	(−3.16, 6.39)
40–49	188	22.6	366	16.4	–		31.0	34.5	–	
50–59	213	25.6	578	26.0	1.39	(0.95, 1.55)	30.5	33.2	0.67	(−3.66, 5.00)
≥ 60	158	19.0	712	32.0	2.31	(1.50, 2.53)	25.7	29.5	−3.72	(−7.95, 0.52)
Nationality										
German	705	84.7	2114	95.0	–		29.5	33.0	–	
Other	127	15.3	112	5.0	0.33	(0.25, 0.44)	38.1	35.3	6.66	(0.36, 12.96)
Examination program baseline										
Level 1	668	80.3	1747	78.5	–		29.9	33.2	–	
Level 2	164	19.7	479	21.5	1.15	(0.93, 1.42)	29.8	33.4	0.38	(−3.01, 3.78)
Reminder baseline										
0	338	40.6	1211	54.4	–		26.3	29.8	–	
1	266	32.0	633	28.4	0.68	(0.55, 0.84)	33.3	34.6	7.80	(4.44, 11.16)
2	128	15.4	252	11.3	0.59	(0.45, 0.77)	31.5	36.1	5.94	(1.27, 10.61)
≥ 3	100	12.0	130	5.8	0.39	(0.29, 0.53)	44.3	43.7	18.52	(12.47, 24.58)
Appointments baseline										
1	586	70.4	1642	73.8	–		30.0	34.1	–	
2	175	21.0	436	19.6	0.84	(0.68, 1.03)	29.1	30.7	−0.51	(−4.02, 2.99)
≥ 3	71	8.5	148	6.6	0.67	(0.49, 0.92)	31.4	30.6	1.94	(−3.67, 7.56)
Phone status baseline										
No phone	689	82.8	1774	79.7	–		30.3	33.7	–	
Phone	143	17.2	452	20.3	0.73	(0.57, 0.93)	28.5	31.2	5.21	(1.39, 9.03)
N	832	27.21	2226	72.79			2226			

^a^Adjusted for duration between baseline examination and follow-up invitation in years and day of the week the invitation were sent

Adding a flyer to the invitation was also not associated to the time to response in days (β̂ = 1.71, 95% CI: − 1.01, 4.44; see Table 2). Persons with non-German nationality took more time to send back the questionnaire (β̂ = 6.66, 95% CI: 0.36, 12.96). If a person needed to be reminded before eventually participating in the baseline examination, it took them also longer to return the completed questionnaire (1 reminder: β̂ = 7.80, 95% CI: 4.44, 11.16; 2 reminders: β̂ = 5.94, 95% CI: 1.27, 10.61; ≥3 reminders: β̂ = 18.52, 95% CI: 12.47, 24.58). Person whose phone numbers had been available before baseline recruitment also responded later β̂ = 5.21, 95% CI: 1.39, 9.03).

## Discussion

The current trial did not provide evidence that an informational flyer, intended as motivation, influenced the likelihood of participation or the time it took participants to return the filled questionnaire. It is important to note that this result only relates to the effectiveness of this particular flyer and especially does not rule out that, with a multitude of possible variations in content and design, other flyers could be more successful in increasing response. Nevertheless this study is a good example on how small evaluation trials can be used to assess the effectiveness of new recruitment measures and information materials, thereby providing information to improve the efficient allocation of resources. For instance, this finding will inform our decision should the situation arise that adding the flyer would raise letters into the next postage tier.

It should raise more concerns, however, that the intensity of recruitment during baseline was associated with participation in the follow-up and that in a negative way. Persons who had to be reminded more often during baseline, or needed more appointments until eventually attending the examination completed the follow-up questionnaire less often and did so more slowly. Hence, the long term benefits of additional efforts to increase response during baseline appear to be limited. And those who responded more slowly at follow-up again caused additional recruitment effort as compared to early respondents, because they received additional reminders and/or needed to be called-up more often according to the recruitment protocol. This finding was corroborated by evidence suggesting that the availability of phone numbers prior to baseline recruitment was associated with a lower likelihood of participation and a slower response. It is known that recruitment by phone results in higher response proportions as compared to sole postal recruitment [17, 18], that is, some persons got convinced to participate at baseline during the phone call that otherwise would have not, and these persons were more reluctant to participate in the follow-up. Note that recruitment by phone is also more time-consuming for the field staff as compared to postal recruitment.

Although our results suggest that additional efforts and resources spent on recruitment during baseline were punished at the follow-up, these findings need careful interpretation. First it is important to note that the relation between recruitment effort at baseline and participation at follow-up is not causal, but rather both variables are dependent on a common cause, that is, traits in the particular individual to be recruited. Consequently, these findings should not lead to decide against more intense recruitments during baseline in order to avoid recruiting participants that are more likely to drop out later on. Not only is low participation a problem already, this strategy might introduce bias by systematically missing out on a sub-population that potentially is different from other participants [9, 10, 19–21]. On the contrary, these results suggest that information about baseline recruitment (i.e., paradata) might be useful to tailor follow-up recruitments to those who were difficult to recruit during baseline, by, for instance, scheduling more and earlier calls for them, offering them better incentives, or, if scientifically justiciable, provide them with less arduous questionnaires (i.e., short forms) [22].

To this end, however, it is necessary to routinely and meticulously collect paradata during recruitment, which not only depends on suitable software, but also on motivated field staff actually taking advantage of the possibilities offered by such software, as this task requires extra effort and diligence. But with such paradata at hand, it is possible to routinely evaluate recruitment measures [14] or utilize responsive recruitment protocols that can reduce non-response bias or increase response by adapting to special sub-populations or to conditions encountered in the field [22].

Motivating individuals to enroll in cohort studies and stay enrolled thereafter is one of the main challenges for population based research [5]. A considerable amount of research focused on low-level technical results of the delivery of invitations and the use of material incentives [6], but there is also research indicating that some participants are motivated by non-material reasons. In addition to their desire to learn more about one’s own health status and receiving personal medical advices, people state as their reasons for enrollment their support for scientific progress, the prospect of gaining insights into research practice, and their trust in the institutions that conduct the research [23, 24]. The use of informational flyers is one reasonable way to convey messages on scientific progress, insights into research practice, and an image of trustworthiness and the flyer evaluated in this trial contained such information. Our results, however, suggest that it did so in an ineffective way and indicate that further research in this area is warranted.

Known limitations of this study include that the trial was only conducted in one of the 18 study centers across Germany due to logistic reasons. Furthermore, the participants included in this study do not constitute a true random sample, because, in order not to disrupt the standardized recruitment protocol for the follow-up questionnaire, they had to be included in order of their invitation. And, as mentioned before, conclusions concerning the effectiveness of informational materials for recruitment are limited to the particular flyer under investigation, which also was designed from the perspective of public relation experts, rather than based on a scientific theory.

## Conclusion

Assessing the effectiveness of new measures and materials utilized for recruitment can provide information to efficiently allocate resources. Paradata collected during baseline recruitment for a cohort study can help identifying subgroups that are less likely to participate in follow-up examinations and therefore could be used to tailor follow-up recruitment protocols accordingly.

## Supplementary information

**Additional file 1.**

## Data Availability

Data analyzed for the current study are not publicly available due to privacy concerns, but will be made available upon reasonable request.

## Abbreviation

GNC: German National Cohort
