# Supplementary material for: Participants who were difficult to recruit at baseline are less likely to complete a follow-up questionnaire – results from the German National Cohort
Source: BMC Med Res Methodol. 2020 Jul 9;20:187. doi: 10.1186/s12874-020-01073-0 (PMC7346423; doi:10.1186/s12874-020-01073-0)

**Supplementary material**

**Participants who were difficult to recruit at baseline are less likely to complete a follow-up questionnaire – Results from the German National Cohort**

**Stefan Rach, Kathrin Günther and Birte Hadeler**

**Contents**

1. Translation of the informational flyer „NAKO update“ (Pages 1 – 2)
2. Flyer “NAKO Update” (German) (Pages 3 – 4)

The order of English translations corresponds to the order of occurrence of the texts in the flyer

| **Why is a follow-up questionnaire on health status conducted after 2-3 years?**  Some people may ask themselves this question. For the scientific validity of the NAKO Health Study it is very important to track changes of the health status of participants. To reliably investigate associations between exposures and diseases it is necessary to collect information about lifestyle, exposures, and diseases not only at one point in time, but also information about new health incidents has to be collected. This includes, for instance, newly diagnosed medical conditions like those under investigation in the NAKO.  Many of the planned scientific analyses will compare different groups of participants. For instance, participants who did not have a certain medical condition at the baseline examination will be compared to other participants who also did not have this medical condition at baseline, but had it diagnosed afterwards. Therefore it is important for the success of this study to collect information about your health via questionnaire, even if no changes as compared to the baseline examinations have occurred. |
| --- |

| **Hand grip strength**  All participants of the NAKO Health Study had their hand grip strength measured three times with each hand. Often participants asked “Why is this measurement taken?” and “What is the meaning of a high or low result?”.  Hand grip strength is a very good indicator for overall muscle strength, that is, on average people with high hand grip strength tend to be stronger in general. The assessment of hand grip strength is much simpler and consistent as compared to the measurement of the strength of other muscles (for instance, the thigh). A small device, called dynamometer, is all you need. Hand grip strength is usually measured in kilogram. In general, maximum hand grip strength is reached about at 40 years of life and this appears also to be true for the data collected in NAKO so far. Mean hand grip strength is 48 kg for males and 30 kg for females. The highest measurement taken so far was 89 kg. |
| --- |

| **Assessing olfaction: the “smell test”**  Olfaction is located in the biologically oldest part of the brain. In the early times of human development it served an important function. It warns of dangers due to gas or fire, helps in the search for water and food, and is important to tell apart edible from inedible foods. Already in newborns olfaction is completely matured. It helps them right after birth to find their mothers breasts. In the first three years, the olfactory memory is established. During this period a large part of olfactory sensations are encountered for the first time and leave their marks in the brain.  Olfaction across the lifespan  With increasing age olfaction declines. Starting with about 40 years of life the ability to detect and differentiate smells declines. Smoking and frequent contact to chemicals, dirt, or dust can also impair olfaction.  Furthermore, certain medical conditions acquired during the lifespan can cause impaired olfaction. There also exist rare congenital olfactory medical conditions.  The olfactory test conducted in NAKO is a so-called “screening test”, that is, a test to detect olfactory impairments/defects. Since the impairment of olfaction can serve as early marker for some neurological conditions, the scientists in NAKO want to investigate olfaction. |
| --- |

| **Change of name: “National cohort” is now “NAKO Health Study”**  The first participants got to know this study as “National cohort” and may now wonder why the name was changed to “NAKO Health Study”. The reasons for this are rather simple to explain. For scientists, the former name was obvious and innocuous, because   1. “National” signified that the 18 study centers are distributed all across Germany and that all participants would represent the whole of the German population – comparable with the German national sport teams; 2. “Cohort” is a scientific technical term for a specific type of study.   But the scientists did not foresee that the term “cohort” could lead to irritation and misunderstandings. Some people, for instance frequent readers of the “Asterix” comics and former students of Latin, connected this term with the military context. Actually the term cohort stems from the Latin name for a formation of soldiers of the Roman army (1/10^th^ of a Legion).  Today the term “cohort” is also common in other contexts -from animal breeding to sociology- and is used for groups selected according to specific criteria and investigated for a specified period of time. |
| --- |


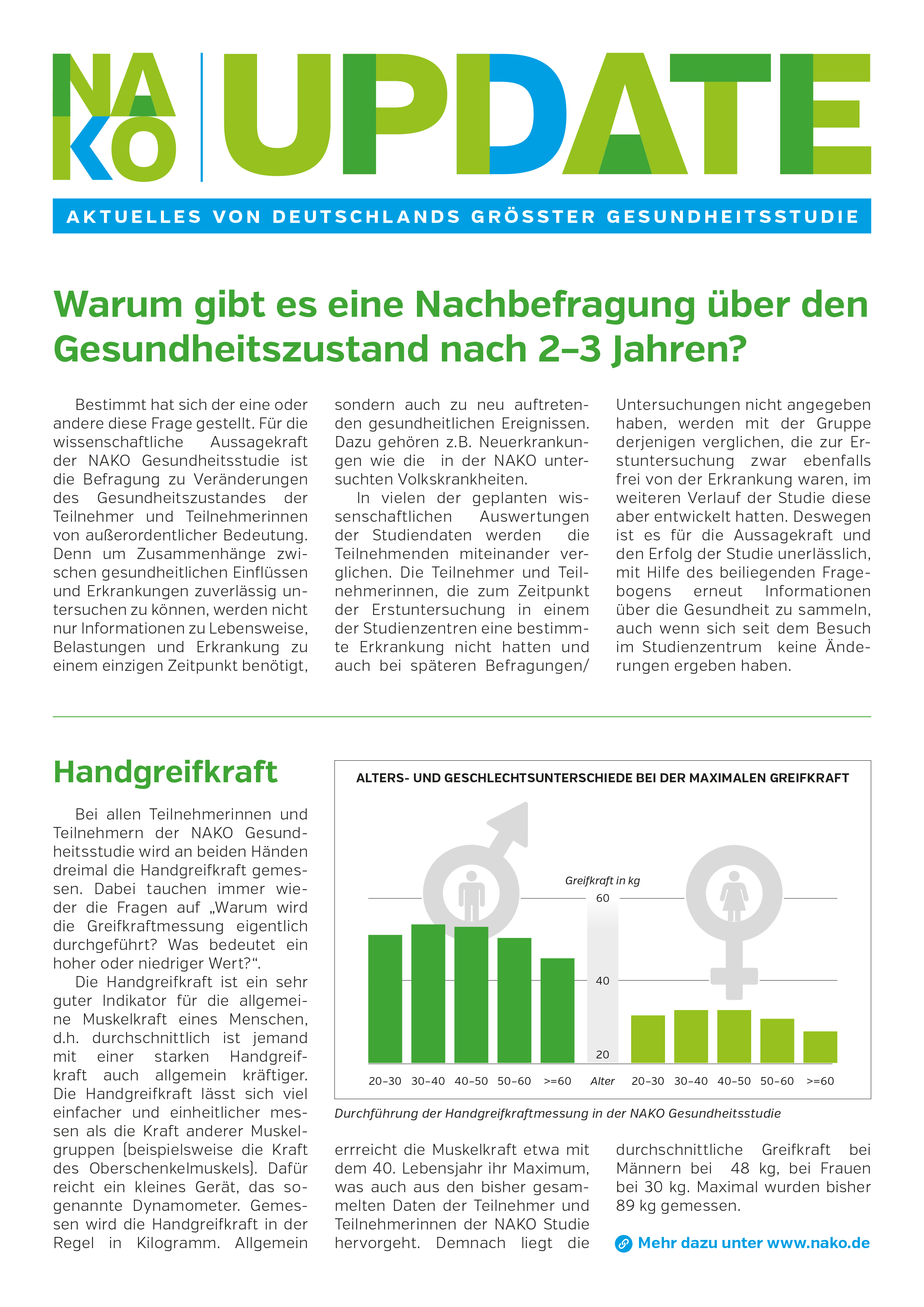

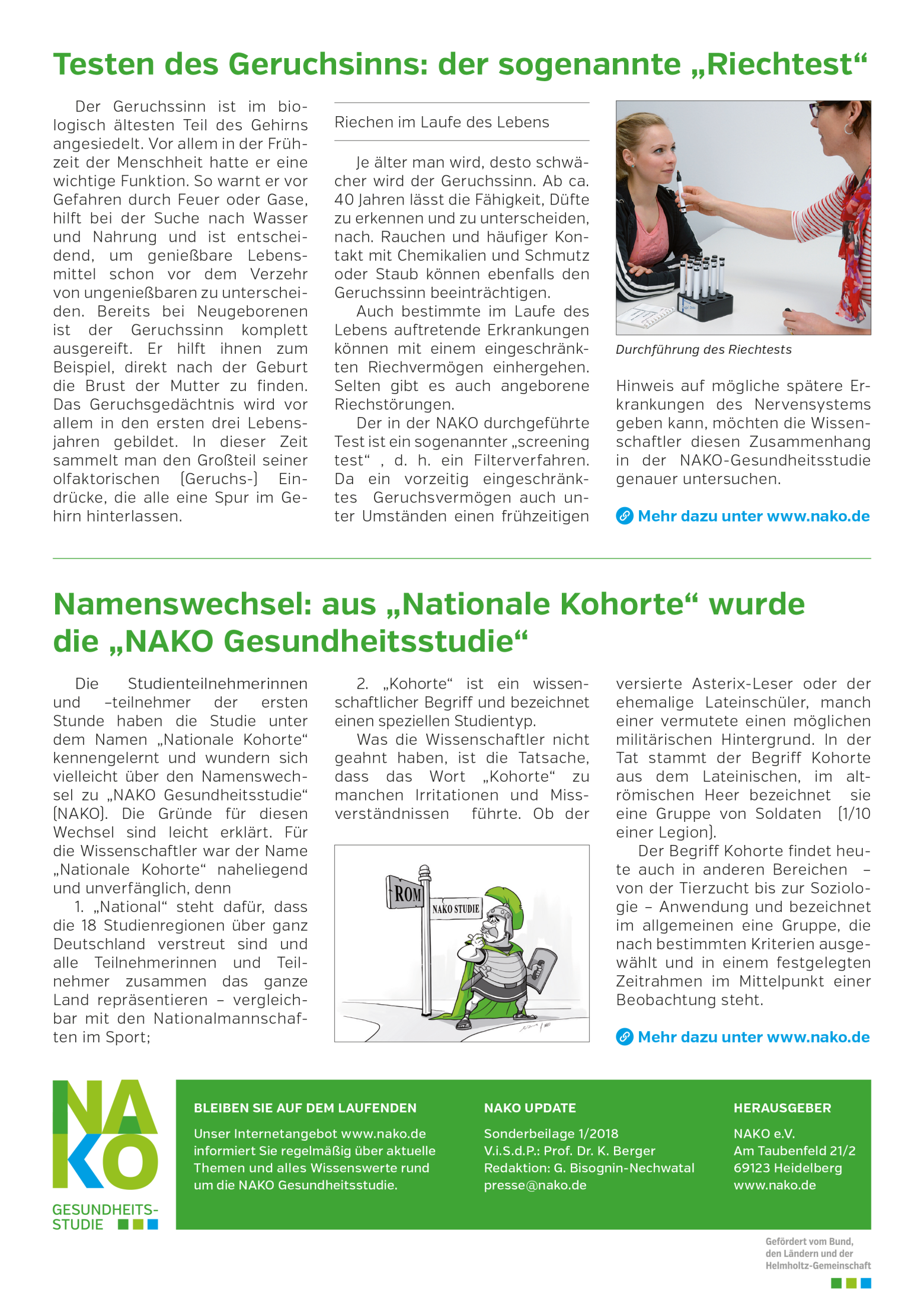

Supplement: Supplementary file 1 — Additional file 1. [file 12874_2020_1073_MOESM1_ESM.docx]
